# Q-switched pulse operation in erbium-doped fiber laser subject to zirconia (ZrO_2_) nanoparticles-based saturable absorber

**DOI:** 10.1016/j.heliyon.2024.e24478

**Published:** 2024-01-14

**Authors:** Umer Sayyab Khalid, Haroon Asghar, Hafsa Hameed, Muhammad Sohail, Adnan Khalil, Rizwan Ahmed, Zeshan A. Umar, Javed Iqbal, M. Aslam Baig

**Affiliations:** a National Centre for Physics, Quaid-i-Azam University Campus, 45320, Islamabad, Pakistan; b Department of Physics, University of Azad Jammu and Kashmir, Muzaffarabad, 13100, Azad Kashmir, Pakistan; c International Collaborative Laboratory of 2D Materials for Optoelectronics Science and Technology of Ministry of Education, Institute of Microscale Optoelectronics, College of Electronics and Information Engineering, Shenzhen University, Shenzhen, 518060, China; d Institute of Physics, Khwaja Fareed University of Engineering and Information Technology, Rahim Yar Khan, Pakistan

**Keywords:** Erbium-doped fiber lasers, Q-switched, Zirconia, Saturable-absorber

## Abstract

In this paper, the zirconia (ZrO_2_) nanoparticles-based saturable-absorber (SA) have been incorporated in an erbium-doped fiber laser (EDFL) cavity for achieving a Q-switched pulse operation. The implementation of the zirconia nanoparticles-based powder on the fiber facet was accomplished using the index-matching gel's adhesion effect. The incorporation of SA in the laser cavity yielded a stable and self-starting Q-switched operation under 19.36 mW pump power that corresponded to the emission wavelength of 1557.29 nm. Additionally, it was observed that the EDFL's emission wavelength tuned from 1557.29 nm to 1562.3 nm , and the repetition rates and pulse width ranged from 61.2 to 130 kHz and 7.9 to 3.6 μs, respectively, as the pump power was increased from 19.36 to 380.16 mW. Measured experimental results reveal that at a maximum pump power of 380.16 mW, the maximum average output power, peak power, and pulse energy were noticed to be 1.17 mW, 2.5 mW, and 9 nJ, respectively. A 52 dB suppression in side bands was found at a pump power of 380.16 mW. Moreover, the stability and threshold tolerance of the EDFL has also been discussed in detail. These findings suggest that nanoparticle-based saturable absorbers have potential applications in a pulsed source, making it easier to implement in fiber cavity-based systems.

## 1 Introduction

Q-switched fiber lasers have attracted significant applications in recent decades including optical communication [1], fiber-based sensing [2], and LIDAR applications [3]. These fiber lasers can operate in both pulsed as well as in continuous-wave (CW) mode operation. In contrast to CW erbium-doped fiber lasers (EDFLs), pulsed EDFLs offer the advantage of producing ultrashort optical pulses of significant peak power by using the mechanism of Q-switching [4] or mode-locking [5]. Various techniques have been adopted to achieve Q-switched pulse operation in fiber lasers. The classification of techniques utilized for Q-switching can be broadly divided into two distinct groups, namely passive and active. The implementation and feasibility of the passive Q-switching technique exceed the active technique in terms of its simplicity, efficiency, and versatility [6]. Passive Q-switching can be achieved through the use of nonlinear polarization rotation (NPR) [7] or by using a saturable absorber (SA) within the cavity arrangement. Several types of SAs have already been demonstrated, such as semiconductor saturable absorber mirrors (SESAMs) [8], carbon nanotubes [9], and two-dimensional materials [10]. When the laser power increases from a certain threshold, its absorption coefficient increases dramatically and the absorber becomes saturated. This leads the laser towards switching of laser output from a CW state to a pulsed state. Recently, several efforts have been dedicated towards the fabrication of SAs through the utilization of chemical vapor deposition [11], liquid-phase-epitaxy [12], nano-powder [[13], [14], [15]], solution methods [16], pulsed laser deposition-based thin films [[17], [18], [19]] and then incorporating the nanostructures or thin-film material within the laser cavity which has significantly enhanced the performance and efficacy of pulsed fiber laser sources.

Most recently, substantial work has been reported on the fabrication of a new type of fiber zirconium oxide–erbium co-doped fiber (Zr-EDF) which acts as a unidirectional amplifying medium [[20], [21], [22]]. The Zr-EDF exhibits non-linear properties including Four-Wave Mixing (FWM) which is an ideal candidate to overcome phase-matching complexities [23]. Zirconium-oxide (ZrO_2_) nanoparticles with their excellent chemical and physical properties like optimum durability and maximum toughness [24], made it an ideal candidate to use as a SA in EDFL to attain a Q-switched pulse operation. ZrO_2_ including a band gap of 3.1 eV [25] present in an optical fiber to enhance the Er and Er/Yb solubility which results in high-power optical amplifier efficiency [26]. A Q-switched EDFL subject to zirconium-disulfide-SA has been proposed and demonstrated. It was investigated that at 345 mW of pump power, the system yields a maximum pulse repetition rate of 87.1 kHz, a shortest pulse width of 1.49 μs, and a maximum pulse energy of 33.5 nJ [27]. Most recently, a zirconium selenide (ZrSe_2_) SA-based EDFL has been demonstrated which is capable of generating 16.75 mW output power, 1.27 ps of pulse duration, and pulse energy of 3.75 nJ [28].

Here, we illustrate a straightforward method for depositing zirconia nanoparticles onto a fiber facet to act as a SA. By utilizing this technique, a zirconia-SA-based EDFL system yields Q-switching at a low pump threshold power of 19.36 mW. Furthermore, the investigation demonstrates that by utilizing a pump power of 380.16 mW, a pulse repetition rate of 130 kHz, a pulse width of 3.6 μs, an average output power of 1.17 mW, a pulse energy of 9 nJ, and peak power of 2.5 mW was obtained.

## 2 Preparation and characterization of zirconia (ZrO_2_)

**Fig. 1:**
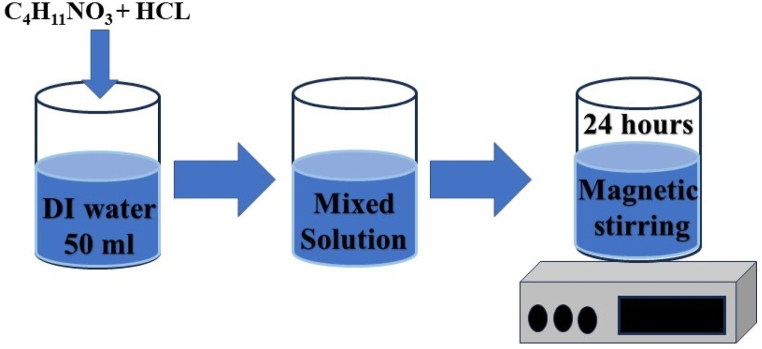
Synthesis process of zirconia nanostructures.

The zirconia nanostructures were synthesized using an approach reported in Ref. [29]. The zirconia nanoparticles were synthesized by combining 0.6 g of C_4_H_11_NO_3_ and 0.3 ml of HCl in 50 ml of deionized water. After the homogeneous mixing, the continuous stirring was performed for 24 h. The formed sample was then collected after centrifugation at 3000 rpm and was washed using deionized water. The nanostructures were finally formed after drying from 24 h. The process of nanostructures prepared using this solution method technique is illustrated in Fig. 1.

**Fig. 2:**
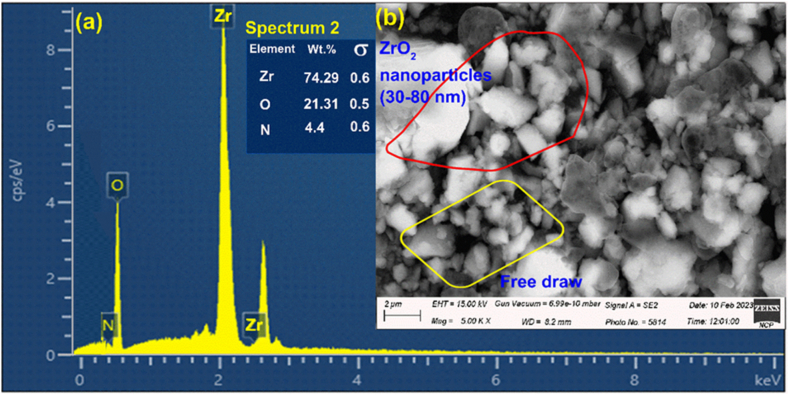
(**a**) EDX spectrum of the ZrO_2_ sample; (**b**) SEM image depicting the surface structure of the ZrO_2_ sample.

An energy-dispersive X-ray spectroscopy (EDX) technique was implemented to investigate the elemental concentration of a ZrO_2_ sample. A sample under investigation was subject to a study of its chemical composition using an Oxford-Instruments X-MAX-N-20 EDX attached with a SEM operating at 30 keV. Fig. 2(a) shows the results of an EDX analysis on a ZrO_2_ sample, which revealed that the sample is mostly made up of Zr (74.29 %), O (21.31 %), and N (4.4 %). The structural characteristics of the ZrO_2_ specimen were assessed through the utilization of scanning electron microscopy (SEM) as illustrated in Fig. 2(b). The micrograph is presented at a resolution of 2 μm which indicates the morphology and structure of ZrO_2_ nanoparticles.

**Fig. 3:**
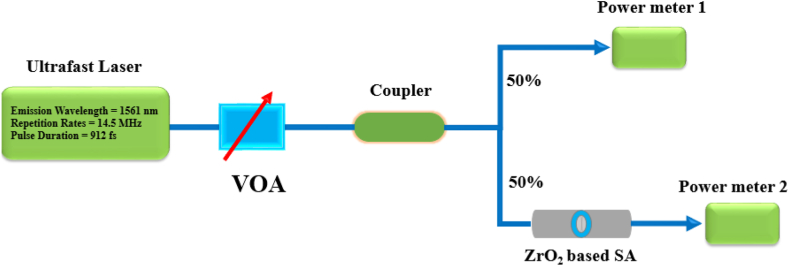
The schematic experimental arrangements for measurement of the nonlinear saturable absorption curve based on zirconia-SA.

**Fig. 4:**
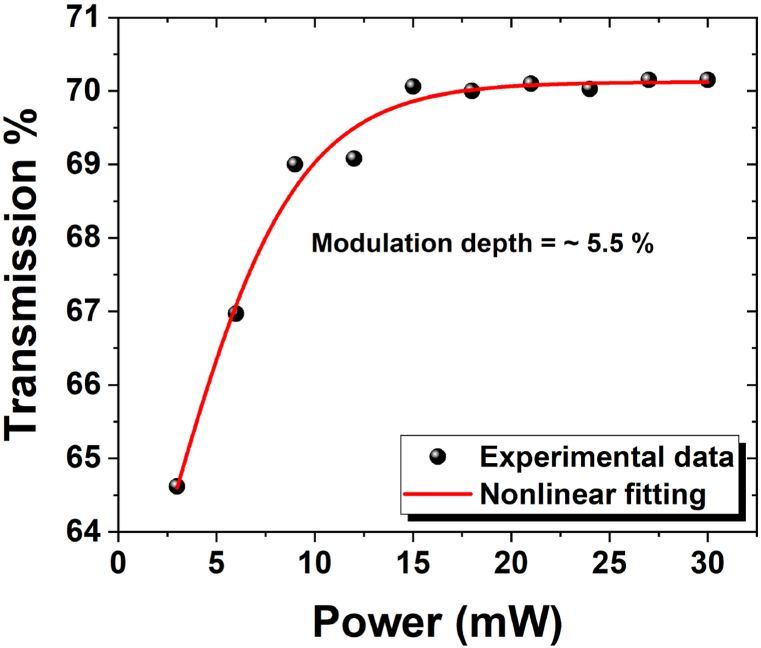
A nonlinear transmission curve as a function of pump laser for zirconia-SA at 1560 nm wavelength source.

To further explore the optical characteristics of zirconia-SA, the non-linear absorption properties are measured by a balanced twin-detector method [30]. The schematic of the experimental arrangement used to measure non-linear absorption properties is illustrated in Fig. 3. In this experiment, a standard femtosecond laser emitting at 1561.2 nm of wavelength, with a repetition rate of 14.5 MHz, and a pulse duration of 912 fs was used. The strength of optical light was controlled using an optical attenuator and then further divided into two parts using the 3-dB coupler. Two power meters were used to measure the optical power; from first, light enters directly, and the second power meter collects light when SA is incorporated into the path. The measured experimental data (solid black circles) and non-linear fitting (solid red line) results are shown in Fig. 4.

A following saturable absorption model was implemented to fit the measured experimental data.

$$T\left(I\right)=1-\Delta T*\exp\left(-\frac{I}{I_{sat}}\right)-T_{ns}$$

Here, *T(I), ΔT, I, I*_*sat*,_ and *I*_*ns*_ represent transmittance, modulation depth, input intensity, saturation intensity, and non-saturation losses, respectively. The modulation depth from the nonlinear transmission curve is measured to be 5.5 %. This indicates that zirconia-based SA can be effectively used for ultrafast pulse generation.

## 3 Experimental setup of the erbium-doped fiber laser

**Fig. 5:**
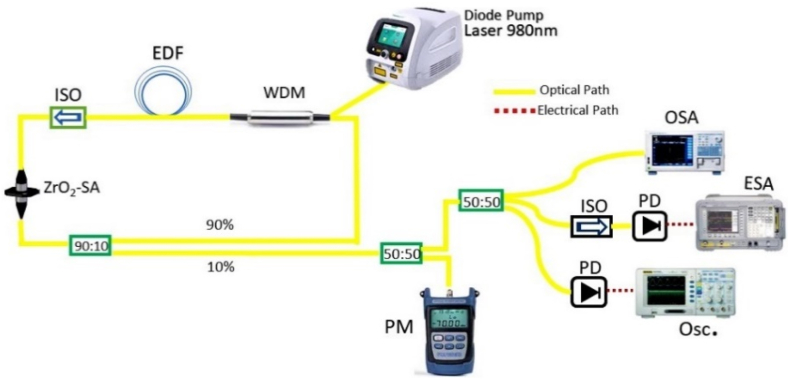
The experimental arrangements of a Q-switched erbium-doped fiber laser based on ZrO_2_-SA.

A schematic of the experimental configuration for the EDFL system including ZrO_2_-SA is presented in Fig. 5. A diode laser, which is pumped continuously at 980 nm, is connected to a doped fiber via a wavelength division multiplexer (WDM) that operates at 980/1550 nm. The gain medium consists of 10 m EDF, with a signal absorption coefficient of 6 dB/m at 1530 nm. Besides the overall length of the cavity was estimated around 12 m. An optical isolator is attached in the fiber-based ring cavity after the doped fiber, which enables the unidirectional flow of optical signals. The ZrO_2_-SA material is positioned between the two fiber ferrules to facilitate interaction with the optical signal. The output power of the EDFL is divided into two parts containing a 90/10 ratio, where 90 % of the signal is redirected into the laser ring cavity and 10 % is reserved for EDFL characteristic analysis. The RF and optical spectra were obtained through a 5 GHz InGaAs photodiode (Thorlabs) using an RF spectrum analyzer (GW INSTEK, GSP-9330) and an optical spectrum analyzer (YOKOGAWA, AQ6370D). In addition, the characteristics of the optical pulse train were conducted utilizing a digital oscilloscope (GW INSTEK, GDS-3504).

## 4 Results and discussions

### 4.1 Q-switched erbium-doped fiber laser subject to ZrO_2_-SA

**Fig. 6:**
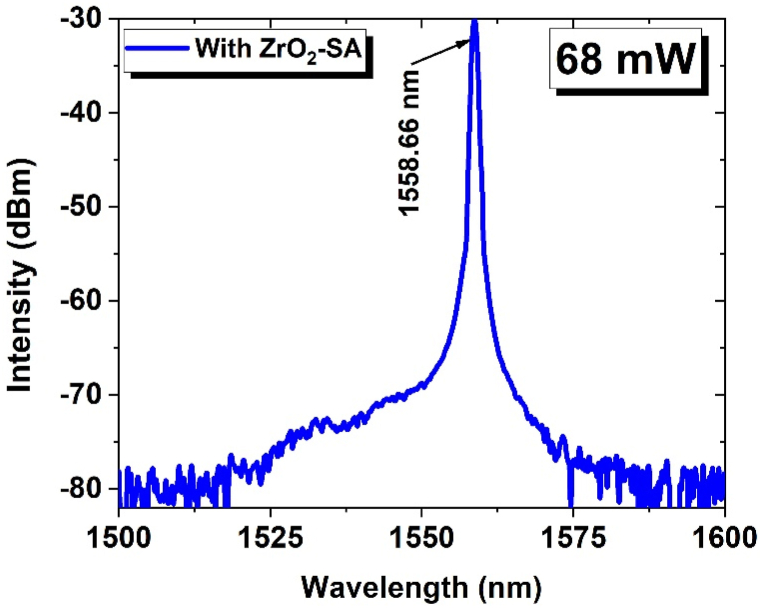
The optical spectra measured at 1558.66 nm using SA based on ZrO_2_ nanoparticles.

The CW operation of an EDFL was successfully obtained up to a pump power of 19.36 mW. Beyond this threshold, a self-starting Q-switched operation was achieved. The emission spectrum of the laser, with the implementation of a SA within the laser cavity, is depicted in Fig. 6. It can be observed that the laser emitted at a 1558.66 nm wavelength. Notably, stable Q-switching was maintained within the pump power range from 19.36 to 380.16 mW. However, beyond the 380.16 mW pump power the Q-switching effect disappeared. It is pertinent to mention here that as the pump power was reduced from 380.16 mW, the Q-switching mechanism was retrieved. These results demonstrate the exceptional thermal stability of the ZrO_2_ nanoparticles-based SA, which exhibits a threshold above 380.16 mW.

**Fig. 7:**
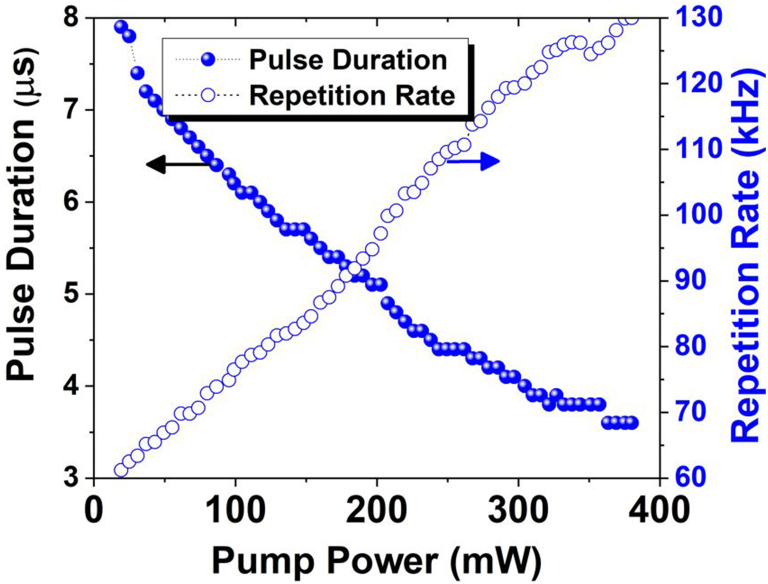
The repetition rate (hollow blue circles) and pulse duration ( solid blue circles) variation versus pump power.

Fig. 7 depicts the pulse width and pulse repetition rates that were measured as a function of the pump, which ranged from 19.36 to 380.16 mW. As the pump rises, there is a corresponding decrease in pulse duration from 7.9 to 3.6 μs and an increase in pulse repetition rate from 61.2 to 130 kHz. The observed trend of increased pulse-repetition rates and reduced pulse-width signifies the distinctive characteristics of Q-switching.

**Fig. 8:**
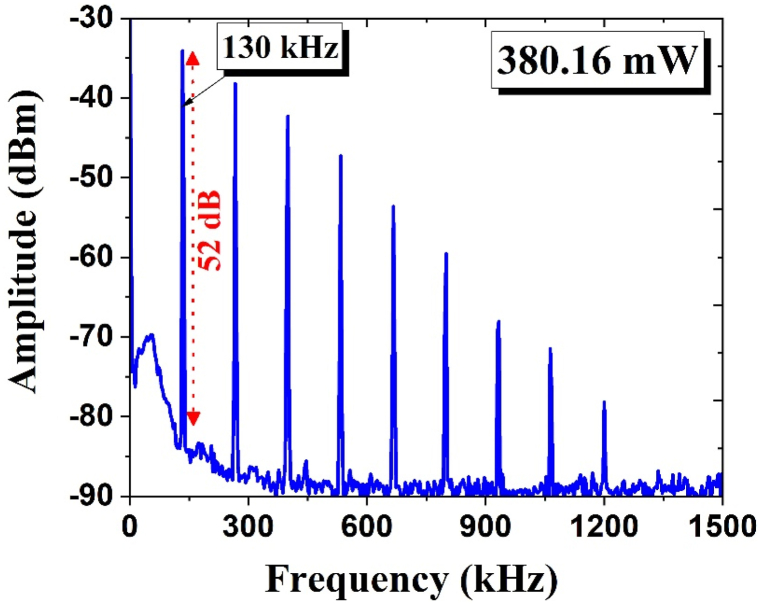
The measured RF spectra of EDFL at 380.16 mW pump power and a 52 dB signal-to-noise ratio.

The RF spectra were recorded at a pump power of 380.16 mW as depicted in Fig. 8, with a frequency range of 1500 kHz, 3 kHz of resolution bandwidth, and a 100 Hz of video bandwidth. The measurements indicate that the fundamental frequency reached 130 kHz with a signal-to-noise ratio (SNR) of 52 dB. A signal-to-noise ratio (SNR) of 52 dB denotes a reliable and stable pulsed laser operation.

**Fig. 9:**
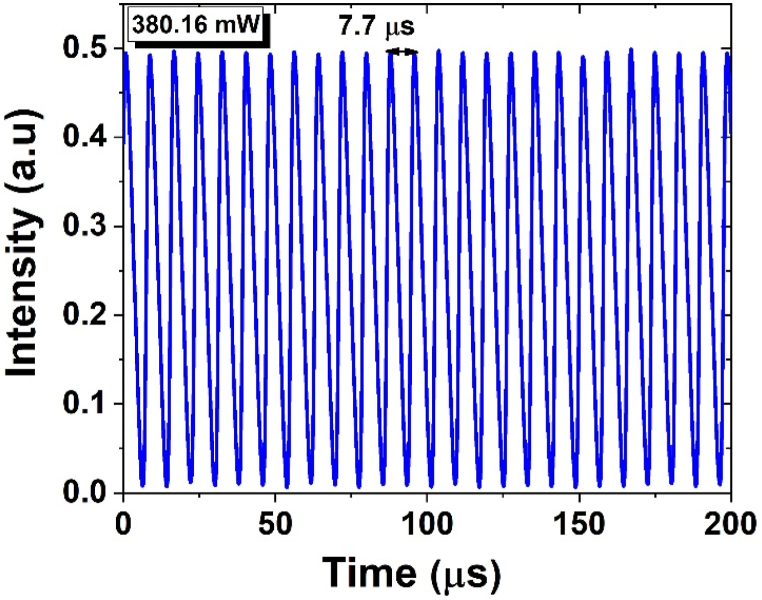
Measured optical pulse trace of an oscilloscope, at maximum pump power of 380.16 mW.

The obtained stable pulse train in our EDFL, depicted in Fig. 9, has a pulse time interval of 7.7 μs that agrees well with the fundamental frequency repetition rate of approximately 130 kHz as shown inFig. 8.

**Fig. 10:**
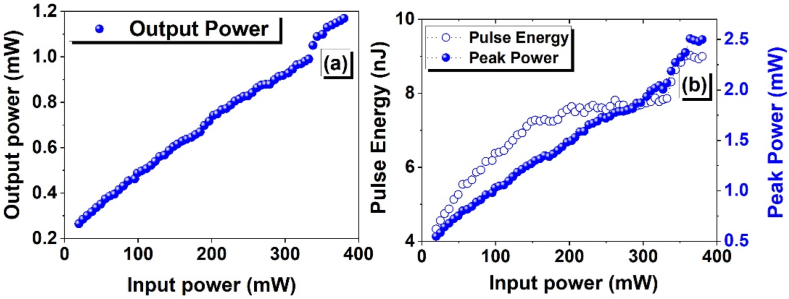
The measured (a) average output power (b) Peak power (solid blue circles) and pulse energy (hollow blue circles) as a result of the pump power ranges from 19.36 to 380.16 mW.

Fig. 10 (a) and (b) illustrate the relation between the output power, peak power, and pulse energy versus the pump power. The findings suggest that the 1.17 mW output power appears at the highest achievable pump power of 380.16 mW. This is accompanied by a corresponding pulse energy of 9 nJ and a peak power of 2.5 mW.

### 4.2 The durability of ZrO_2_-SA for erbium-doped fiber laser

**Fig. 11:**
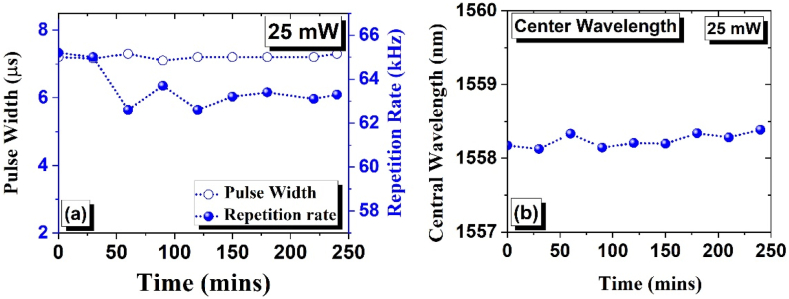
(a) The measured pulse width (hollow blue circles) and repetition rate (solid blue circles) versus pump power (b) The shift in central wavelength of EDFL based on ZrO_2_-SA undergo 30-min intervals over 4 h.

In the following section, the stability of the EDFL was examined based on *ZrO*_*2*_*-SA*. To verify the stability of the EDFL, the SA was subjected to continuous exposure at 25 mW pump power for 4 h. The resulting data on the shift in the pulse duration, center wavelength, and repetition rates is shown in Fig. 11 (a) and (b). The experimental findings indicate a consistent pulse width throughout the 4-hour duration, while the repetition rate shows slight fluctuations. However, it should be noted that only a slight variation in the wavelength was seen as a function of time that validates the stability of the suggested arrangements.

## 5 Conclusion

In summary, we have demonstrated the use of ZrO_2_-SA in a Q-switched Erbium-doped fiber laser (EDFL). The prepared SA has proven to generate Q-switched pulse operation with a wavelength of 1558.66 nm, resulting in higher repetition rates up to 130 kHz, and a pulse duration of 3.6 μs at an available pump power of 380.16 mW. Additionally, the average output power, pulse energy, and peak powers were achieved to be 1.17 mW, 9 nJ, and 2.5 mW, respectively, under similar pump power. Notably, the proposed SA exhibits optimum stability in terms of minor wavelength shift, stable pulse duration, and repetition rates, indicating its potential for generating Q-switched optical pulses in the field of photonics for developing pulsed laser sources.

## Data availability statement

Data will be made available on request.

## CRediT authorship contribution statement

**Umer Sayyab Khalid:** Methodology, Formal Analysis, Investigation, Writing-original draft. **Haroon Asghar:** Conceptualization, Methodology, Writing-original draft, Supervision. **Hafsa Hameed:** Methodology, Formal Analysis, Investigation. **Muhammad Sohail:** Formal Analysis, Investigation. **Adnan Khalil:** Characterizations. **Rizwan Ahmed:** Formal Analysis. **Zeshan A. Umar:** Characterizations. **Javed Iqbal:** Investigation. **M. Aslam Baig:** Writing - review & editing.

## Declaration of competing interest

The authors declare that they have no known competing financial interests or personal relationships that could have appeared to influence the work reported in this paper.
