# Supplementary material for: Q-switched pulse operation in erbium-doped fiber laser subject to zirconia (ZrO2) nanoparticles-based saturable absorber
Source: Heliyon. 2024 Jan 14;10(2):e24478. doi: 10.1016/j.heliyon.2024.e24478 (PMC10828679; doi:10.1016/j.heliyon.2024.e24478)
Supplement: Multimedia component 1 [file mmc1.pdf]

Form to confirm authorship changes for *Heliyon*

This form must be ***signed by all authors*** when there is a change in authorship which includes changes to any of the following items: author name(s), order of the authors, the corresponding author(s), the addition of authors, the removal of authors and changes in affiliation.

By personally signing this note, **all** authors confirm that: I) the changes are in accordance with their scientific contribution, II) they agree with all the changes and III) confirm that the authorship list conforms to the authorship criteria outlined on Heliyon's ethics page. IV) it is the responsibility of the corresponding author to get the signature from all co-authors accepting the change. In case of any ethic violation/malpractice in the signature, the corresponding author is accountable. The completed form should be returned along with the final/revised manuscript to proceed further with the manuscript. Manuscripts for which incomplete forms have been submitted will be rejected within 5 working days.

Any disputes on the authorship list and contributions need to be resolved by the involved scientists and *Heliyon* will only proceed with the evaluation of the manuscript once we receive confirmation, through this form, that such an agreement between the authors has been reached.

***Heliyon* will not accept changes to the authorship list in the late stages of the editorial process (when a paper is in Accept in Principle stage, acceptance or after publication)**

Manuscript number: HELIYON- D-23-47211R1

Article title: Q-switched pulse operation in erbium-doped fiber laser subject to Zirconia (ZrO_2) based Saturable-Absorber

Complete new author list: Umer Sayyab Khalid, HAROON Asghar, Hafsa Hameed, Mohammad Sohail, Adnan Khalil, Rizwan Ahmed, Z. A. Umar, Javed Iqbal and M. Aslam Baig

Date: 27-12-23

Scanned with CamScanner

#	First name	Last name	Dept. & institution name	Institutional email address	Order change (Y/N)	Addition/Deletion	Change in Author name	Affiliation Change (Y/N)	Reason for change	Signature
1	Umer Sayyab	Khalid	*[1]National Centre for Physics, Quaid-i-Azam University Campus, 45320 Islamabad, Pakistan [2]Department of Physics, University of Azad Jammu and Kashmir, Muzaffarabad 13100, Azad Kashmir, Pakistan*	umer.sayyab33@gmail.com	N		N	N		
2	Haroon	Asghar	National Centre for Physics, Quaid-i-Azam University Campus, 45320 Islamabad, Pakistan	haroon.asghar92@gmail.com	N		N	N		
3	Hafsa	Hameed	National Centre for Physics, Quaid-i-Azam University Campus, 45320 Islamabad, Pakistan	hafsa.hameeda@yahoo.com	N		N	N		Hafsa Hameed
4.	Muhammad	Sohail	*International Collaborative Laboratory of 2D Materials for Optoelectronics Science and Technology of Ministry of Education, Institute of Microscale Optoelectronics, College of Electronics and Information Engineering, Shenzhen University, Shenzhen, 518060, China*	sohailphys76@gmail.com	N		N	N		Sohail.
5	Adnan	Khalil	*Institute of Physics, Khwaja Fareed*	adnan.khalil@uftu.edu.[illegible]	Y		N	N	Contribution	

			University of Engineering and Information Technology, Rahim Yar Khan, Pakistan							
6	Rizwan	Ahmed	National Centre for Physics, Quaid-i-Azam University Campus, 45320 Islamabad, Pakistan	rizwan.ahmed@ncp.edu	Y		N	N	Contribution	
7	Zeshan A.	Umar	National Centre for Physics, Quaid-i-Azam University Campus, 45320 Islamabad, Pakistan	zadael@ncp.edu	Y		N	N	Contribution	
8	Javed	Iqbal	*Department of Physics, University of Azad Jammu and Kashmir, Muzaffarabad 13100, Azad Kashmir, Pakistan*	Javed.iqbal@ajku.edu.pk	Y		N	N	Contribution	
9	M.Aslam	Baig	National Centre for Physics, Quaid-i-Azam University Campus, 45320 Islamabad, Pakistan	baig.m@ncp.edu	Y		N	N	Contribution	
10.	Tahan. A.	ALzeba	Princess Nora binte Abdul Rehman Riyadh, Saudi Arabia	taalzebi@pn.edu	Y	Deletion	N	N	Not enough contribution	
